# Responding to rapid change in libraries: a user experience approach

**DOI:** 10.29173/jchla29616

**Published:** 2022-08-04

**Authors:** Jessica McEwan

**Affiliations:** MLIS, User Experience Librarian University of Ottawa, Ottawa, ON Canada

Bignoli, C & Stara, L. **Responding to rapid change in libraries: a user experience approach**. Chicago: ALA Editions; 2020. Softcover: 136 p. ISBN: 978-0-8389-4835-4. Price: USD$44.99. Available from: https://www.alastore.ala.org/content/responding-rapid-change-libraries-user-experience-approach

Early in this book, authors Bignoli and Stara quote web designer and writer Kissane, who compares online information that requires tending and care to remain usable to a live green plant. Bignoli and Stara think of libraries and their collections as living plants, too, and ask readers to see their book’s fundamental argument (that change is constant) in this plant image: we should expect our library (aka our plant) to grow and change. In embracing that change is constant, we can affect development in planned, intentional ways; we can work to better our understanding of our plant and adjust for its needs; and we can prune with intention and make changes over time in small increments. In short, this book aims to help the reader cultivate library services through a continued and purposeful process of questioning and planning, with consideration for the needs of users.

Bignoli and Stara draw on their professional experience as they walk us along this adventure of embracing change in library services management. Bignoli is a director of a small engineering college library in New England with experience in public, academic, and U.S. state-level libraries while Stara is an architect and librarian also situated in the U.S., specializing in library building design. Both have authored content on user experience (UX) methods, and both contribute anecdotes from their professional work to enliven the text. It will come as no surprise that this work does not speak directly to health librarianship or a healthcare setting, and in parts it is quite U.S. centric. However, this does not detract from the book’s message or its practical approach. I believe those who are new to managing small-scale library systems, are solo librarians in corporate or embedded libraries, or have responsibility for public services and spaces will find this book most relevant to their work.

Over seven chapters, the reader is given pragmatic cues for tackling change piece by piece. Users at the center of everything (chapter two) introduces baseline UX terminology and ideas and gives examples of places to start when improving the library experience for users. Library technology (chapter four) is the longest in the book and looks at technologies that form the backbone of a modern library. This includes integrated library systems, catalogue interfaces, and discovery layers, but also looks beyond these to the question of how point-of-sale systems and self-checkout terminals developed for commercial enterprise can be adapted for the library environment. Physical spaces (chapter five) examines collection development policies, types of accessibility to consider with physical space design, signage, wayfinding systems, and planning for flexibility rather than renovation as a long-term approach to managing future space needs. This is a taste of the book’s content but is by no means exhaustive.

Each chapter has a list of references, and the text is peppered with relevant or amusing graphics and images. Throughout, the reader is encouraged to think about the people who will use the library and its services and to step away from the status quo by asking: Is there a good reason for this?

For me, some of the most interesting reading was in the opening chapter, Customer service expectations for the twenty-first century. Here the authors explore how customer experiences provided by dominant technology and retail corporations impact the modern library. Looking outside the library world, the authors model how to look for clues about current service expectation in research conducted by service organizations with analogous touchpoints. Concepts such as self-service options, frictionless experiences, and service quality expectations are explored through research conducted for airline and hospitality industries as examples.

It would be easy to close this book and conclude that adopting a user mindset, or thinking like a user, is a lot of what UX is about. The authors often urge us to think like a user and the term “user experience” is right there in the book's title after all. There are suggestions to conduct focus groups, talk to patrons, or perhaps do some interviews. Yet the mechanisms for undertaking this type of work are not addressed beyond general references to current voices in UX (such as Norman, Schmidt and Etches, Steve Krug, and IDEO).

Discussing this aspect with a colleague experienced in library UX, Friberg’s recent paper on leadership in UX came up where Friberg points to a subtle but important difference between assuming a user experience perspective (i.e., thinking like a user) and observing and listening to a user's lived experience (i.e., UX research). Thinking like a user can be risky business: it brings a library into a closed circuit of thinking that can serve to perpetuate the status quo. If no new information comes into the circuit, how can the library better understand its users? On the other hand, learning about the user’s lived experience through UX research that is designed to observe and listen to users allows a library to set aside status quo thinking and move forward with new information as a guide for planning services and spaces.

As my colleague said in reflecting on her own UX work over the years, “I'm STILL surprised by people.” Indeed, for colleagues working in health librarianship with its evidence-based culture, Friberg’s argument that evidence trumps assumption should resonate.

Many valuable ideas and strategies are presented in this book, among them embracing uncertainty, consideration for user experience, the value of customer research in fields with common touchpoints, and library management in a rapidly evolving customer service ecosystem. I would encourage my colleagues, though, to not stop here. Partner this reading with additional reading on introductory UX research methods and use these techniques with the ideas in Bignoli and Stara’s text to bring new information about your own users to bear on local change management.
